# FusoPortal: an Interactive Repository of Hybrid MinION-Sequenced *Fusobacterium* Genomes Improves Gene Identification and Characterization

**DOI:** 10.1128/mSphere.00228-18

**Published:** 2018-07-05

**Authors:** Blake E. Sanders, Ariana Umana, Justin A. Lemkul, Daniel J. Slade

**Affiliations:** a Department of Biochemistry, Virginia Polytechnic Institute and State University, Blacksburg, Virginia, USA; University of Kentucky

**Keywords:** *Fusobacterium*, *Fusobacterium nucleatum*, pathogenesis, colon cancer, colorectal cancer, genomics, host-pathogen

## Abstract

Here we present FusoPortal, an interactive repository of *Fusobacterium* genomes that were sequenced using a hybrid MinION long-read sequencing pipeline, followed by assembly and annotation using a diverse portfolio of predominantly open-source software. Significant efforts were made to provide genomic and bioinformatic data as downloadable files, including raw sequencing reads, genome maps, gene annotations, protein functional analysis and classifications, and a custom BLAST server for FusoPortal genomes. FusoPortal has been initiated with eight complete genomes, of which seven were previously only drafts that ranged from 24 to 67 contigs. We have showcased that the genomes in FusoPortal provide accurate open reading frame annotations and have corrected a number of large (>3-kb) genes that were previously misannotated due to contig boundaries. In summary, FusoPortal (http://fusoportal.org) is the first database of MinION-sequenced and completely assembled *Fusobacterium* genomes, and this central *Fusobacterium* genomic and bioinformatic resource will aid the scientific community in developing a deeper understanding of how this human pathogen contributes to an array of diseases, including periodontitis and colorectal cancer.

**IMPORTANCE** In this report, we describe a hybrid MinION whole-genome sequencing pipeline and the genomic characteristics of the first eight *Fusobacterium* strains deposited in the FusoPortal database. This collection of highly accurate and complete genomes drastically improves upon previous multicontig assemblies by correcting and newly identifying a significant number of open reading frames. We believe that the availability of this resource will result in the discovery of proteins and molecular mechanisms used by an oral pathogen, with the potential to further our understanding of how Fusobacterium nucleatum contributes to a repertoire of diseases, including periodontitis, preterm birth, and colorectal cancer.

## Abstract (precis)

In this report, we describe a hybrid MinION whole-genome sequencing pipeline and the genomic characteristics of the first eight *Fusobacterium* strains deposited in the FusoPortal database. This collection of highly accurate and complete genomes drastically improves upon previous multicontig assemblies by correcting and newly identifying a significant number of open reading frames. We believe that the availability of this resource will result in the discovery of proteins and molecular mechanisms used by an oral pathogen, with the potential to further our understanding of how Fusobacterium nucleatum contributes to a repertoire of diseases, including periodontitis, preterm birth, and colorectal cancer.

## INTRODUCTION

Multiple *Fusobacterium* species are oral pathogens that infect a broad range of human organ and tissue niches (1, 2). Fusobacterium nucleatum has recently been connected with colorectal cancer (CRC) (3, 4), with studies showing that this bacterium induces a proinflammatory microenvironment and chemoresistance against drugs used to treat CRC (5–7). Despite the importance of *Fusobacterium* in human diseases, a lack of complete genomes of biomedically relevant isolates has hindered protein cataloging and virulence factor identification. Many bacterial draft genomes have been sequenced and partially assembled using short-read technologies (Illumina, 454 Life Sciences), leaving complete genome assembly difficult due to the presence of repeat regions. The reference genome of F. nucleatum subsp. nucleatum ATCC 25586 was completed using cosmid and λ phage technologies to achieve long (10-to-35-kb) cosmid insertions and whole-genome assembly (8). However, we show in a parallel report that whereas this genome was assembled into one complete chromosome, we uncovered a 452-kb inversion using our sequencing and assembly methods. This inversion does not appear to change many open reading frames, but it could be useful in whole-genome comparisons to analyze detailed gene orientations. With the emergence of next-generation long-read sequencing (Pacific Biosciences, Oxford Nanopore MinION), assembling whole genomes has become standard practice and affordable for academic research settings. The recent combination of MinION long-read and Illumina short-read technologies to scaffold and polish DNA sequencing data, respectively, has created a robust pipeline for bacterial genome completion and subsequent gene identification and characterization (9, 10).

The motivation for complete sequencing and assembly of *Fusobacterium* genomes came from our discovery that bioinformatic analysis identified a high percentage of large genes (~3,000 to 12,000 bp) in the F. nucleatum subsp. nucleatum ATCC 23726 genome that appeared to correspond to proteins missing critical domains at either the N or C terminus (e.g., >2,000-amino acid deletions). We showed that these genomic discrepancies are not isolated to F. nucleatum subsp. nucleatum ATCC 23726 by correcting a substantial number of large proteins (up to 5,300-amino-acid corrections) encoded in all eight genomes. Since the largest proteins in *Fusobacterium* are autotransporters of the type 5 secretion system, these new genomes will be important to reevaluate the virulence factor landscape of these pathogenic bacteria.

To provide ease of use and data accessibility to the community, we have used this study to launch the FusoPortal repository (http://fusoportal.org), which provides the first eight completely sequenced, assembled, and annotated *Fusobacterium* genomes using MinION and Illumina technology. While databases such as KEGG, NCBI, and UniProt are crucial for researchers to find open reading frames, our goal was to create a central database in which researchers interested in *Fusobacterium* biology could obtain high-quality data in an easy-to-navigate platform. The FusoPortal repository framework has been developed to allow additional genomes to efficiently be added, with the goal of assembling 25 previously incomplete genomes spanning a broad range of *Fusobacterium* species. In summary, genomes and bioinformatic analyses available in the FusoPortal repository provide key resources to further determine how this understudied pathogen contributes to a variety of human infections and diseases. Here we highlight not only how users can interact with the FusoPortal website but also additional bioinformatic analysis that was made possible by improved genome sequencing and assembly.

## RESULTS AND DISCUSSION

### Genome sequencing, assembly, and annotation.

**FIG 1:**
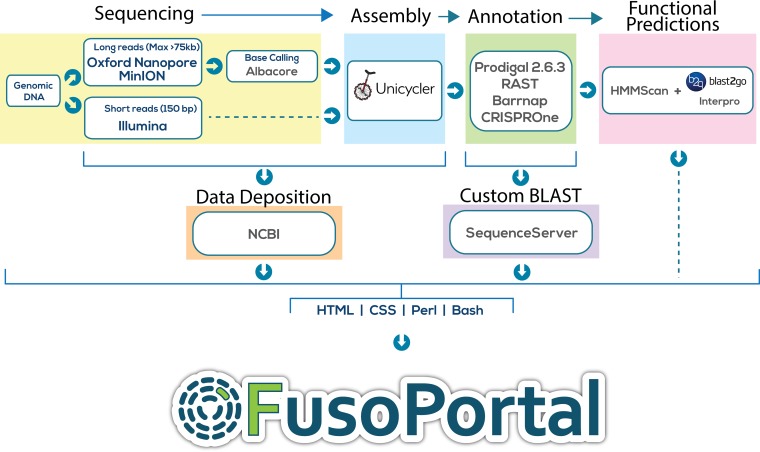
Schematic of all genomic, bioinformatic, and scripting workflows used to create FusoPortal.

In this study, we successfully completed seven new *Fusobacterium* genomes and resequenced the widely referenced strain F. nucleatum subsp. nucleatum 25586. Each genome now consists of a single circular chromosome, and in the case of F. varium 27725, we report the discovery of a 42-kb circular plasmid that contains 70 genes encoding proteins ranging in size from 38 to 1,678 amino acids. For each genome, we have provided access to all protein- and RNA-encoding genes and have highlighted the previously unidentified CRISPR systems. In Fig. 1, we describe the workflow used to complete genome sequencing, assembly, annotation, functional prediction, and implementation of the interactive FusoPortal repository. We made a concerted effort to use open-source software when possible, to make this workflow reproducible and accessible to the scientific community.

**FIG 2:**
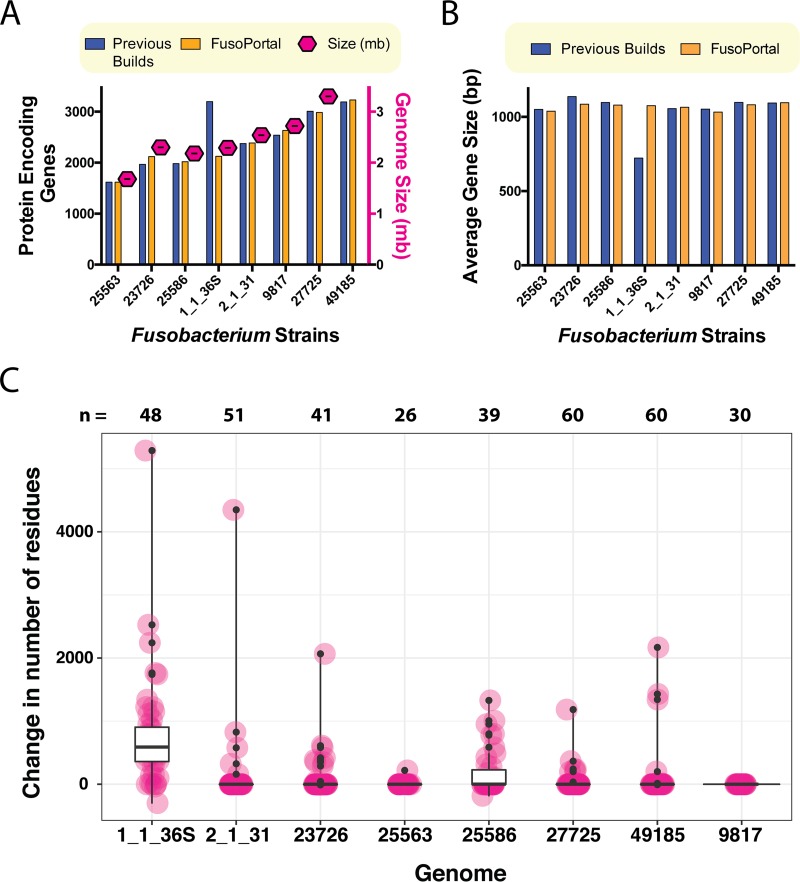
Correction of open reading frames in *Fusobacterium* genomes. (A) A comparison of the genome size to the number of protein-encoding genes per genome in both the NCBI and FusoPortal genomes. (B) A comparison of all proteins for average gene size in the NCBI and FusoPortal genomes. (C) Analysis of all proteins 1,000 residues in size and above in annotated FusoPortal proteomes and of how many residues were added (or, in rare cases, removed) compared to the previous annotations present in the NCBI database. n* =* number of proteins 1,000 residues in size or greater in each genome.

We highlight that the strain with the largest change in gene number from a reference genome to our single-chromosome build was F. necrophorum *funduliforme* 1_1_36S, which was previously reported with 3,197 protein-encoding genes. We analyzed our new prediction of 2,125 genes and show that this number is much more consistent with the genome size seen in comparisons of all *Fusobacterium* genomes (Fig. 2A) (Table 1). These data show that the previous annotation contained an abundance of short open reading frames and that the complete genome increases the average gene length by more than 250 bp; the average gene size of ~1,000 bp agrees well with the data for the remaining seven *Fusobacterium* genomes (Fig. 2B).

**TABLE 1:** Statistics and NCBI accession numbers for FusoPortal genomes

Species	Strain	NCBI GenBank sequence accession no.	No. of NCBI contigs^a^	Size (Mb)	No. of protein genes	FusoPortal GenBank sequence accession no.	No. of FusoPortal contigs	Size (Mb)	No. of protein genes
F. nucleatum	23726	GCF_000178895.1	67	2.23	1,983	GCA_003019785.1	1	2.30	2,111
F. nucleatum	25586	GCA_000007325.1	1	2.17	1,968	GCA_003019295.1	1	2.18	2,019
F. varium	27725	GCA_000159915.2	39	3.30	3,008	GCA_003019655.1	1	3.35	3,054^b^
F. ulcerans	49185	GCA_000158315.2	49	3.49	3,191	GCA_003019675.1	1	3.54	3,230
F. mortiferum	9817	GCA_000158195.2	44	2.67	2,538	GCA_003019315.1	1	2.72	2,631
F. gonidiaformans	25563	GCA_000158835.2	24	1.70	1,618	GCA_003019695.1	1	1.68	1,617
F. periodonticum	2_1_31	GCA_000158215.3	61	2.55	2,375	GCA_003019755.1	1	2.54	2,388
F. necrophorum	1_1_36S	GCA_000242215.1	40	2.31	3,197	GCA_003019715.1	1	2.29	21,215

a Contig, continuous stretch of genomic DNA with breaks.

b Data include 70 genes from a 42-kb plasmid (GCA_003019655.1).

### Correcting large protein-encoding reading frames.

As expected, the largest change in the F. necrophorum *funduliforme* 1_1_36S genome is in the number of genes that are now annotated with 1,000 amino acids or more. In fact, the combined analyses of all eight genomes show that we have corrected a significant percentage of proteins that were previously annotated with <1,000 amino acids and are now annotated with >1,000 amino acids or of proteins that were already annotated with >1,000 amino acids and are now annotated with expanded protein open reading frames (Fig. 2C). The most extreme example was a previously annotated protein encoded in the F. necrophorum *funduliforme* 1_1_36S genome that was 960 amino acids in size (EHO18576.1) and that has now been annotated as a 6,248-amino acid protein in FusoPortal. F. mortiferum 9817 did not have any amino acid additions in proteins over 1,000 residues in size, but we note that the largest protein encoded in this genome consists of 1,602 residues (EEO35225.2), and as expected, proteins in this range have fewer open reading frame errors than the larger proteins frequently found in *Fusobacterium* genomes. We highlight that in the biomedically significant and genetically tractable strain F. nucleatum subsp. nucleatum ATCC 23726, several large stretches of amino acids were added to proteins that are homologous to the previously characterized virulence protein Fap2 (11–13). In addition, we corrected an abnormally large number of genes in the reference strain F. nucleatum subsp. nucleatum ATCC 25586, which we attribute to improved bacterial annotation software and not to genomic errors in the previous complete genome.

### Whole-genome phylogenetic analysis of a diverse group of *Fusobacterium* spp.

**FIG 3:**
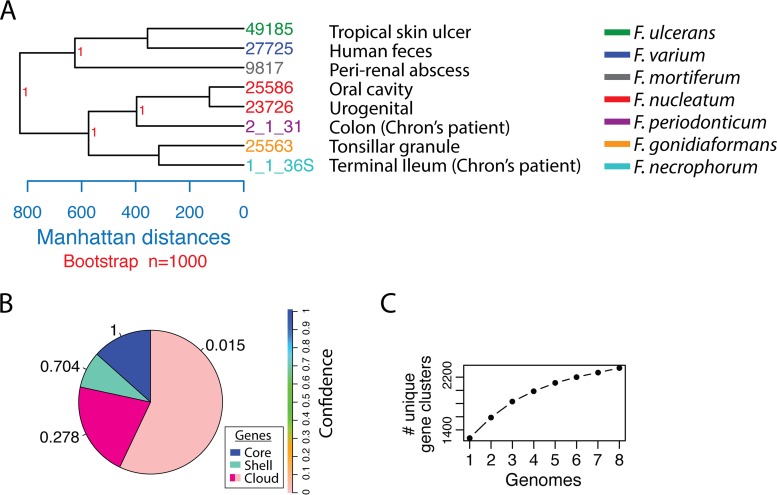
Phylogenetic analysis of complete *Fusobacterium* genomes. (A) Tree created using complete proteomes in the microPan package in R. Bootstrap values are indicated in red. Locations of where strains were isolated in the human body are highlighted. (B) Micropan analysis of pan-genome gene families with models predicting the percentages of core genes, shell genes (present in most genomes), and cloud genes (found in few genomes). (C) Analysis of the number of unique gene clusters found among the eight complete FusoPortal genomes.

With newly identified genes in our reported *Fusobacterium* genomes, we set out to create a phylogenetic tree to compare to previous reports. By using all open reading frames from each genome to build a tree, we show that while F. nucleatum subsp. nucleatum ATCC 23726 and F. nucleatum subsp. nucleatum ATCC 25586 are quite close phylogenetically, the remaining genomes are quite diverse in their genetic makeup (Fig. 3). Because of the diversity of these eight genomes, there are a large number of unique gene clusters which had been analyzed previously and were hypothesized to govern differences in intracellular invasion and virulence potential (14). We highlight the site of isolation of each strain in Fig. 3A as described by Manson-McGuire et al. (14) and anticipate that as we complete more genomes to expand the phylogenetic analysis this tree may provide more extensively detailed insight into genotype-specific populations associated with tissue distribution and human diseases.

### Features of the FusoPortal repository.

**FIG 4:**
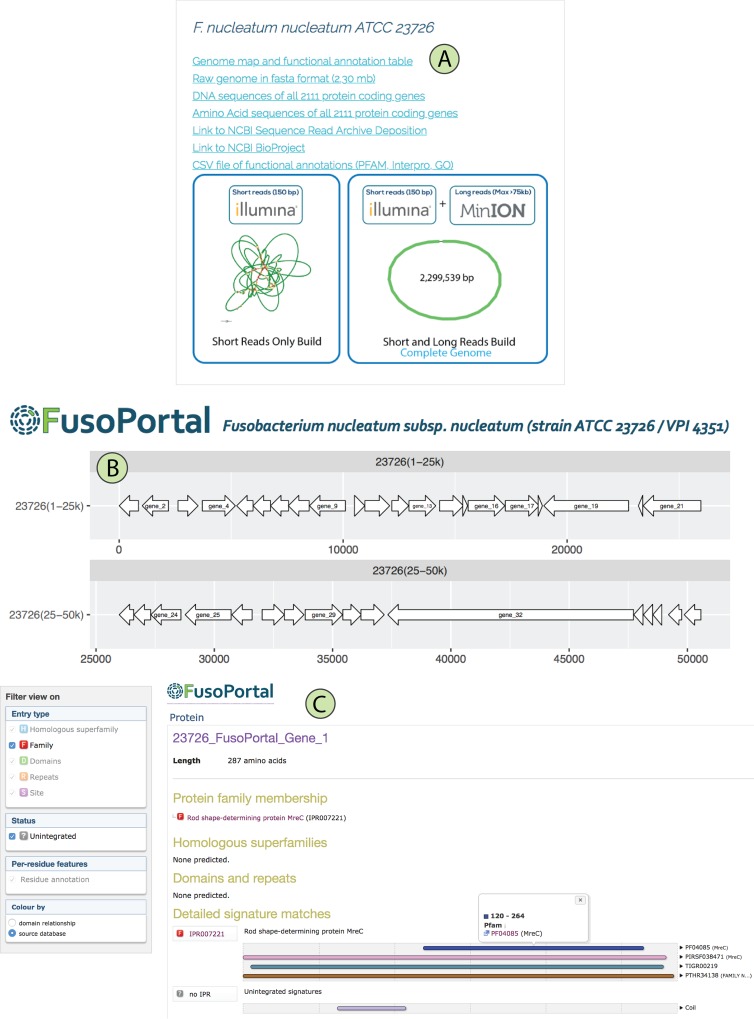
Navigating the FusoPortal Web interface for genome analysis. (A) Links are provided for downloading all genomic data (raw and analyzed), with Bandage plots to show the completeness of each genome build. (B) Whole-genome maps were produced with links to custom webpages for each gene that provide access to all genomic and bioinformatic analysis. (C) Each gene contains a full InterPro analysis page with links to multiple functional prediction databases (e.g., PFAM, TIGR, Gene3D, CDD, GO). No IPR, no InterPro accession number.

FusoPortal was built on an HTML5 framework and is therefore functional on all full-size computers and mobile devices. The home page gives a description of features available in FusoPortal and provides links to all genomes and bioinformatic data. Links to all raw (Illumina and MinION DNA reads) and processed genomes and annotations are available as shown in Fig. 4A. In Fig. 4B, we highlight a genome map in which directional arrows represent clickable open reading frame designations that send the user to individual pages containing DNA and protein coding sequences in FASTA format. We have also analyzed all proteins on FusoPortal using HMMer (15) (downloadable .out files) and have produced custom linked InterPro pages with full bioinformatic analyses (Fig. 4C). These resources alleviate the need for users to exit the site to acquire bioinformatic data and provide functional predictions for entire proteomes.

### A custom BLAST database to search FusoPortal.

**FIG 5:**
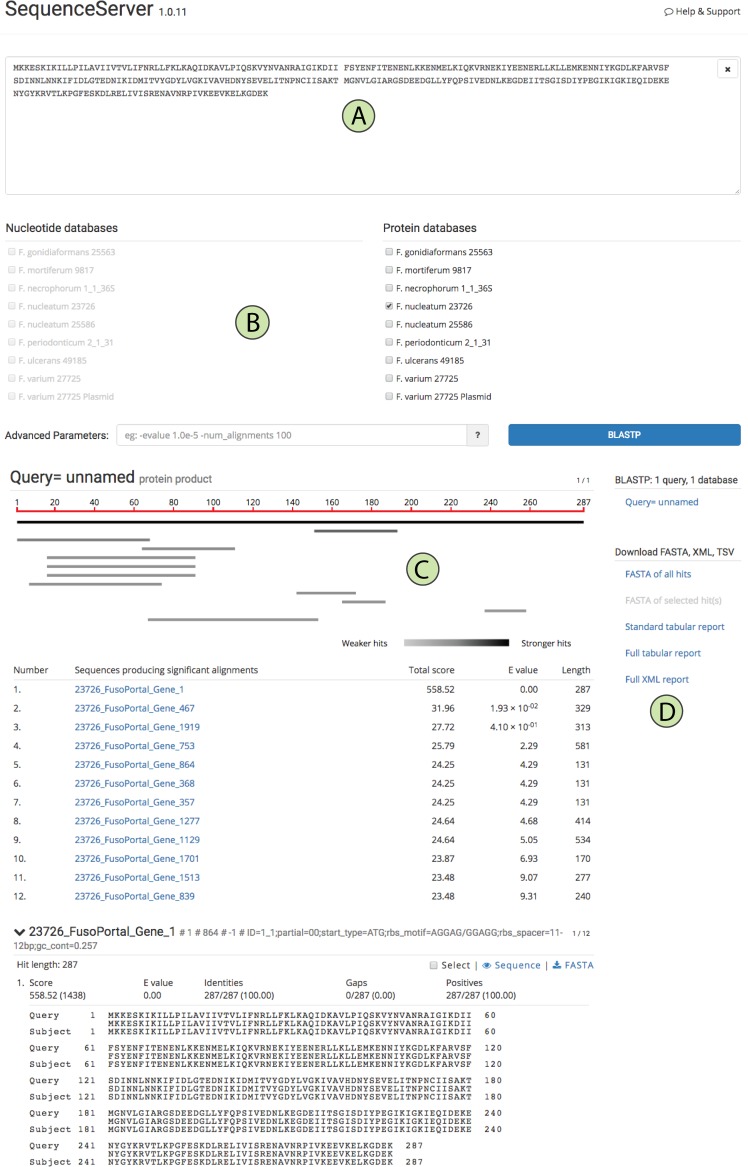
A custom BLAST database to search FusoPortal genomes built with SequenceServer. (A) Data entry panel that autodetects protein or DNA sequences. (B) The available genomes that users can choose to search. (C) BLAST results for identified genes. (D) Links to downloadable files.

To additionally aid in virulence factor identification, we built a custom BLAST server using the open source software Sequenceserver (16). All eight genomes, including the F. varium 27725 plasmid, can be searched using a DNA or protein sequence input as shown in Fig. 5A and B. Results are provided as alignments with E values (customizable inputs for thresholds), and all acquired sequences and alignments can be downloaded in various formats (Fig. 5C and D).

### Conclusion.

FusoPortal is a database of fully sequenced, annotated, and bioinformatically characterized *Fusobacterium* genomes that provides a central location to increase our understanding of these virulent bacteria, which contribute to a wide range of diseases. At this time, we are keeping FusoPortal as a resource that is populated only with genomes sequenced and assembled in the laboratory of D. J. Slade. However, we are open to accepting requests to sequence and deposit additional *Fusobacterium* strains that are of value to the scientific community. Currently, FusoPortal excels at providing genomes that are accurate in both their sequences and their gene annotations. Our goal was to create a clean user interface that allows users to find data in an efficient manner compared with larger databases with steeper learning curves. The introduction of a custom BLAST server provides an interactive environment to detect specific proteins in *Fusobacterium* genomes. At this time, FusoPortal does not contain as many features as other websites such as the BioCyc database collection, EcoGene, and KEGG websites, but we aim to expand the capabilities provided on FusoPortal with both internal and external contributions. In summary, we have provided FusoPortal as a *Fusobacterium*-specific resource and aim to expand this database for increased efficiency in performing detailed comparative genomics and virulence factor predictions for *Fusobacterium* species.

## MATERIALS AND METHODS

### Data to populate FusoPortal.

Detailed sequencing statistics and assembly methods are reported in a concurrent publication by Todd and colleagues (17). Briefly, genomic DNA was isolated from *Fusobacterium* cultures and sequenced using MinION (Oxford Nanopore Technologies) and Illumina platforms. Genomes were assembled using the open source software package Unicycler version 0.4.3 (9). Gene annotations were obtained using Prodigal version 2.6.3 (18), RAST-SDK version 0.0.12 (via KBase) (19), Barrnap version 0.8 (20), and CRISPRone (21). Prediction of protein functions was achieved with the stand-alone HMMer version 3.1 program (15) and with InterPro (22) from within the Blast2GO software platform (23). A graphical representation of methods for DNA sequencing, genome assembly, bioinformatics, and construction of FusoPortal is presented in Fig. 1. As this resource could require expansion or correction of sequencing or reading frame annotation, we note that are implementing versioning and state on the FusoPortal website that all genomes have been initiated at version 1.

### Development of interactive genome maps.

Protein open reading frames predicted by Prodigal (18) were used to identify boundaries that were used in the R package gggenes to create complete genome maps. For each genome, DNA gene coordinates were placed in a .csv file and imported into the gggenes package in RStudio to create linear genome maps. Maps were then manually loaded into Adobe Illustrator for custom FusoPortal formatting, followed by adding links to genes using Adobe Acrobat Pro. Final genome maps were exported as .pdf files for incorporation into the FusoPortal website. All .csv files with gene coordinates and a template R file are provided on our Open Science Framework database (http://osf.io/2c8pv).

### Construction of FusoPortal using HTML and automated field-filling scripts.

FusoPortal was built using HTML5 and CSS, and automation of HTML page filling with genomic and bioinformatic data was implemented using custom Bash and Perl scripts. All scripts are available on an Open Science Framework database (http://osf.io/2c8pv) run by D. J. Slade. Scripts were developed to automate the acquisition of custom HMMer models for all genomes and to produce linear genome maps using gggenes in the Studio package. For populating FusoPortal HTML pages, scripts were created to extract Prodigal gene coordinates from .gbk files, extract gene and protein sequences, and populate webpages with InterPro functional annotations from .csv files for population into HTML pages. InterPro analysis pages were produced by Blast2GO, and the FusoPortal logo was added to each page through scripting.

### Construction of a FusoPortal custom BLAST server.

The FusoPortal custom BLAST server was built using the Sequenceserver software package (16). Briefly, a custom Apache server was implemented on an Amazon Light Sail private server, and all FusoPortal genomes containing protein open reading frames in DNA (.fna) and amino acid (.faa) formats were implemented as guided by the Sequenceserver advanced setup and configuration documentation. This BLAST server can be accessed at http://18.216.121.101/blast/.

### Accession number(s).

All accession numbers for data are provided in Table 1. In addition, all raw sequencing data have been deposited in the NCBI sequence read archive (SRA) as documented in a companion manuscript accompanying this report (17).
