# Erratum for Sanders et al., “FusoPortal: an Interactive Repository of Hybrid MinION-Sequenced *Fusobacterium* Genomes Improves Gene Identification and Characterization”

**DOI:** 10.1128/mSphere.00379-18

**Published:** 2018-07-25

**Authors:** Blake E. Sanders, Ariana Umana, Justin A. Lemkul, Daniel J. Slade

**Affiliations:** a Department of Biochemistry, Virginia Polytechnic Institute and State University, Blacksburg, Virginia, USA

## ERRATUM

Volume 3, no. 4, e00228-18, 2018, https://doi.org/10.1128/mSphere.00228-18. Page 5 of the PDF, Table 1, last row, last column: “21,215” should be “2,125.”

